# Association of plasma homocysteine with cognitive impairment in patients with Parkinson’s disease

**DOI:** 10.3389/fnagi.2024.1434943

**Published:** 2024-12-09

**Authors:** Yan Xiao, Lin-Hua Gan, Xiao-Niu Liang, Zhi-Heng Xu, Tian-Yu Hu, Xiu-Yuan Li, Yi-Lin Tang, Jian Wang, Yi-Qi Liu

**Affiliations:** Department of Neurology and National Research Center for Aging and Medicine and National Center for Neurological Disorders, State Key Laboratory of Medical Neurobiology, Huashan Hospital, Fudan University, Shanghai, China

**Keywords:** Parkinson’s disease, homocysteine, mild cognitive impairment, cognitive function, executive function

## Abstract

**Background:**

Elevated plasma homocysteine (Hcy) has been reported as a risk factor for cognitive impairment in the general population. However, there are conflicting results regarding the relationship between Hcy and cognitive impairment across various cognitive domains in Parkinson’s disease (PD).

**Objective:**

This study aims to explore the association between plasma Hcy levels, cognitive impairment, and dysfunction in various cognitive domains among PD patients with and without mild cognitive impairment (MCI).

**Methods:**

A total of 101 PD patients underwent plasma Hcy measurement, comprising 50 PD-MCI patients and 51 patients with normal cognition (PD-NC). A battery of neuropsychological tests was administered to assess different cognitive domains. Adjusted generalized linear models were used to assess the correlations between Hcy levels and cognitive functions.

**Results:**

As anticipated, PD-MCI patients demonstrated a significant decline in cognitive function across all five cognitive domains (memory, executive function, attention/working memory, language, and visuospatial function). Elevated plasma Hcy levels (≥ 10 μmol/L) were associated with a higher odds of PD-MCI, even within the normal range of Hcy levels (< 15 μmol/L). After adjusting for confounding factors, a negative correlation was observed between plasma Hcy levels and the performance on specific cognitive tests evaluating executive functions in PD, such as the Stroop Color-Word Test-C (β = −1.123, 95% CI = −1.845 ∼−0.401, *p* = 0.0023).

**Conclusion:**

This study underscores a significant link between plasma Hcy levels and PD-MCI, particularly concerning executive dysfunction, even within the normal range of Hcy levels (< 15 μmol/L).

## Highlights

• The plasma homocysteine (Hcy) level is significantly elevated in PD patients with mild cognitive impairment (PD-MCI).
• Plasma Hcy level is significantly associated with the odds of PD-MCI even within the normal range.
• Elevated plasma Hcy level is particularly correlated with executive dysfunction in PD patients.

## 1 Introduction

Parkinson’s disease (PD) is one of the most common neurodegenerative disorders characterized by motor dysfunction and non-motor symptoms. As the disease progresses, non-motor symptoms such as cognitive impairment, depression, sleep disorders, and autonomic dysfunctions may emerge as significant burdens and become key determinants of the quality of life for PD patients (Schapira et al., 2017). Cognitive impairment is the most prevalent and disabling non-motor symptom in PD, ranging from mild cognitive impairment (PD-MCI) to dementia (PDD) (Jones et al., 2017). During the natural progression of the disease, up to 83% of patients with PD may experience some degree of cognitive dysfunction (Hely et al., 2008). MCI represents a transitional stage between normal aging and dementia (Goldman et al., 2018). A meta-analysis, which included data from over seven thousand PD patients, reported a 40% prevalence of MCI, which could manifest at any time during the disease course (Goldman et al., 2018; Baiano et al., 2020). Despite ongoing efforts to improve early detection and management of MCI, current therapies are limited and primarily focus on symptom relief (Langa and Levine, 2014). Therefore, there is a pressing need to identify modifiable risk factors for this complication.

Homocysteine (Hcy) is an intermediary substance produced within the methionine cycle, which plays a crucial role in maintaining methionine and methylation levels in the human body. Numerous studies have highlighted associations between Hcy and various disorders, including cardiovascular diseases, cancer, and certain neurodegenerative diseases (Zhou, 2023). Elevated plasma Hcy levels, which can be caused by levodopa metabolism and treated with B-vitamin and folic acid, are considered a significant risk factor for PD and cognitive impairment in the general population (Teunissen et al., 2005; Zhou, 2023). However, it has been a topic of investigation for many years with conflicting results regarding the relationship between plasma Hcy levels and cognitive impairment in PD. Some studies have found no association (Hassin-Baer et al., 2006; Camicioli et al., 2009; Rodriguez-Oroz et al., 2009; Song et al., 2013), while others have reported more severe cognitive impairment in PD patients with hyperhomocysteinemia (O’Suilleabhain et al., 2004; Ozer et al., 2006; Zoccolella et al., 2009; Zoccolella et al., 2010; Białecka et al., 2012). Additionally, the link between PD-MCI and Hcy remains unclear. PD-MCI patients often exhibit deficits across multiple cognitive domains, with 65 to 93% experiencing some combination of different cognitive deficits (Goldman et al., 2018). Therefore, investigating the association of Hcy with impairment in various cognitive domains may provide insights into the underlying pathophysiology of PD-MCI.

Given this background, our study aims to explore the association between Hcy levels and cognitive functions in PD patients diagnosed with and without MCI, as well as its relationship to other non-motor symptoms.

## 2 Materials and methods

### 2.1 Study participants

All participants were recruited from the Movement Disorders Clinic, Department of Neurology, Huashan Hospital, Fudan University in Shanghai, China, spanning from December 2017 to November 2022. They all fulfilled the Movement Disorder Society (MDS) Clinical Diagnostic Criteria for idiopathic PD (Postuma et al., 2015). Approval for this study was obtained from the Medical Ethics Committee of Huashan Hospital, Fudan University, Shanghai, China. Written informed consent was obtained from all participants and/or their legal representatives prior to their involvement in the study. The study adhered to the principles outlined in the Declaration of Helsinki.

The demographic characteristics were acquired from the participants and/or proxy through a questionnaire, including age, gender, year of education, disease duration, and onset age. Motor symptoms were assessed by two senior investigators specializing in movement disorders. The severity of motor symptoms was evaluated using the Unified Parkinson’s Disease Rating Scale part III (UPDRS-III), items 18–31, after a minimum of 12 h off anti-parkinsonian medications (OFF state). The disease stage of all patients was determined using the Hoehn & Yahr staging scale (H&Y stage). Additionally, participants completed various scales to evaluate non-motor symptoms, including the Geriatric Depression Rating Scale (GDS), Epworth Sleepiness Scale (ESS), Rapid Eye Movement Sleep Behavior Disorder Screening Questionnaire (RBDSQ), Non-Motor Symptoms Scale (NMSS), 39-item Parkinson’s Disease Questionnaire (PDQ-39), and Sniffin’ Sticks Screening 12 Test (SSST-12), as outlined in our previous study (Fan et al., 2020). The dosage of anti-parkinsonian drugs was converted into a total levodopa equivalent daily dose (LEDD) for standardization of medications (Tomlinson et al., 2010).

Cognitive assessments were conducted on patients while they had received their usual anti-parkinsonian medications (in the ON state). Global cognitive ability was assessed using the Mini-Mental State Examination (MMSE) (Zhang et al., 1990). A battery of neuropsychological tests, as detailed in our previous study (Fan et al., 2020), was employed to evaluate five specific cognitive domains: memory, executive function, attention/working memory, language, and visuospatial function. The PD-MCI diagnosis was determined using the more stringent MDS Task Force Level 2 criteria when the PD patients were visited at baseline.

The peripheral blood samples of all patients were obtained from the medial cubital vein after a 12-h fast, on the day of neuropsychological assessment, and Hcy levels were measured using an enzymatic cycling assay conducted by the clinical laboratory of Huashan Hospital.

### 2.2 Statistical analysis

Continuous variables were presented as means ± standard deviation (SD), while categorical variables were expressed as frequencies (%). The normal distribution of data was assessed using the Kolmogorov-Smirnov test and visual histograms. When the continuous data followed a normal distribution, the Student’s *t*-test was utilized for comparisons between the two groups. The Wilcoxon rank-sum test was applied for data with a non-normal distribution. The Pearson chi-square test was used for categorical variables. The logistic regression model was employed to investigate the association between Hcy and PD-MCI, with the results reported as odds ratio (OR) and 95% confidence interval (CI). Correlations between Hcy levels and non-motor symptoms or cognitive functions were assessed using the generalized linear model (GLM), adjusting for age, gender, education year, disease duration, and LEDD. Two-tailed *p*-values were reported, and statistically significant differences were considered as *p* < 0.05. Data analysis and visualization were performed using SAS 9.4 (SAS Institute Inc., Cary, NC, USA).

## 3 Results

### 3.1 Demographics and clinical characteristics

The study enrolled 101 cases, comprising 50 PD-MCI patients and 51 PD patients with normal cognition (PD-NC). Demographic and clinical details are summarized in Table 1. The mean age of all enrolled patients was 62.59 ± 10.15 years, with a mean age of onset of 58.68 ± 10.39 years and a mean disease duration of 49.49 ± 44.01 months. There were no statistically significant differences between these two groups in terms of gender, age, age of onset, disease duration, LEDD, and H&Y stage, except for the education level, which was comparatively lower in PD-MCI patients (*p* = 0.0419).

**TABLE 1:** Demographic and clinical characteristics of enrolled PD patients.

	Total (*N* = 101)	PD-NC (*N* = 51)	PD-MCI (*N* = 50)	*p*-value
Hcy (μmol/L)	11.41 ± 3.30	9.72 ± 1.86	13.12 ± 3.57	< 0.0001*
Hcy [*n* (%)]				< 0.0001*
< 10 μmol/L	36 (35.64%)	28 (54.90%)	8 (16.00%)	
≥ 10 μmol/L	65 (64.36%)	23 (45.10%)	42 (84.00%)	
Female [*n* (%)]	37 (36.63%)	21 (41.18%)	16 (32.00%)	0.3386
Education (year)	10.01 ± 4.20	10.84 ± 4.52	9.24 ± 3.75	0.0419*
Age of onset (year)	58.68 ± 10.39	58.63 ± 10.42	58.73 ± 10.46	0.9753^#^
Age (year)	62.59 ± 10.15	62.37 ± 10.14	62.82 ± 10.27	0.7967
Duration of disease (month)	49.49 ± 44.01	42.94 ± 39.44	56.49 ± 47.88	0.2105
LEDD (mg)	424.25 ± 262.90	394.84 ± 247.64	456.60 ± 279.34	0.4885
H&Y stage [*n* (%)]				0.2629
1	7 (6.93%)	4 (7.84%)	3 (6.00%)	
2	52 (51.49%)	29 (56.86%)	23 (46.00%)	
3	38 (37.62%)	17 (33.33%)	21 (42.00%)	
4	3 (2.97%)	0 (0.00%)	3 (6.00%)	
5	1 (0.99%)	1 (1.96%)	0 (0.00%)	
**Non-motor symptom assessment**				
BDI	11.75 ± 8.01	10.04 ± 7.48	13.65 ± 8.23	0.0268*
GDS	9.71 ± 6.68	8.43 ± 6.64	11.16 ± 6.49	0.0246*
PDQ-39	32.08 ± 26.52	30.25 ± 29.21	34.11 ± 23.33	0.1168
NMSS	12.05 ± 7.83	11.31 ± 8.44	12.87 ± 7.10	0.2246
ESS	6.36 ± 4.24	6.12 ± 3.98	6.64 ± 4.53	0.5342
RBDSQ	4.32 ± 2.96	4.33 ± 3.18	4.31 ± 2.72	0.7009
SSST-12	5.08 ± 2.32	5.05 ± 2.22	5.13 ± 2.46	0.9963
**Cognitive function assessment**				
MMSE	27.07 ± 2.40	27.51 ± 2.32	26.62 ± 2.42	0.0140*
**Memory**				
AVLT-delayed	3.32 ± 2.62	4.73 ± 2.71	2.69 ± 2.34	0.0039*
AVLT-total	19.83 ± 8.56	23.95 ± 8.09	18.02 ± 8.20	0.0101* ^#^
CFT-delayed	9.10 ± 6.09	11.95 ± 5.27	7.79 ± 6.05	0.0083*
**Executive function**				
SCWT-C (sec)	96.81 ± 33.78	84.19 ± 27.51	102.33 ± 35.02	0.0218*
SCWT-C (score)	42.70 ± 7.40	45.24 ± 6.38	41.58 ± 7.61	0.0173*
TMT-B (sec)	163.88 ± 65.98	134.27 ± 41.23	177.74 ± 71.02	0.0083* ^#^
**Language**				
BNT	21.60 ± 4.44	24.45 ± 3.19	20.34 ± 4.35	0.0002*
AFT	14.35 ± 3.65	16.32 ± 2.19	13.47 ± 3.84	0.0014*
**Visuospatial function**				
CFT	24.99 ± 9.96	32.30 ± 2.99	21.62 ± 10.26	< 0.0001*
CDT	23.00 ± 6.72	25.59 ± 3.83	21.81 ± 7.43	0.0539
**Attention/working memory**				
SDMT	30.04 ± 12.50	38.45 ± 11.76	26.11 ± 10.88	0.0003* ^#^
TMT-A (sec)	73.96 ± 36.48	57.77 ± 19.86	81.38 ± 39.98	0.0077*

Continuous variables are expressed as the means ± standard deviation (SD), and categorical variables are expressed as frequencies (%). The bold *p*-value was statistically significant.

*PD-NC vs. PD-MCI.

^#^The *P*-value was calculated using the Student’s *t*-test; the others were calculated using the Wilcoxon rank-sum test. AFT, Animal Fluency Test; AVLT, Auditory Verbal Learning Test; BDI, Beck Depression Inventory; BNT, Boston Naming Test; CDT, Clock Drawing Test; CFT, Rey-Osterrieth Complex Figure Test; ESS, Epworth Sleepiness Scale; GDS, Geriatric Depression Rating Scale; MMSE, Mini-Mental State Examination; NMSS, Non-Motor Symptoms Scale; PDQ-39, 39-item Parkinson’s Disease Questionnaire; RBDSQ, Rapid Eye Movement Sleep Behavior Disorder Screening Questionnaire; SCWT-C, Stroop Color-Word Test C; SDMT, Symbol Digit Modalities Test; TMT-A & B, Trail Making Test A and B; SSST-12, Sniffin’ Sticks Screening 12 Test; PD-NC, Parkinson’s disease with normal cognition; PD-MCI, Parkinson’s disease with mild cognitive impairment; LEDD, levodopa-equivalent daily dose; H&Y stage, Hoehn & Yahr-staging-scale; Hcy, homocysteine.

As shown in Table 1, PD-MCI patients exhibited a statistically significant decrease in MMSE score (*p* = 0.0140), along with notably elevated BDI and GDS scores (*p* = 0.0268 and 0.0246, respectively). Analysis across the five cognitive domains showed a substantial decrease in memory, executive, language, visuospatial, and attention/working memory functions in PD-MCI patients, except for the clock drawing test (CDT) assessing visuospatial function.

### 3.2 Comparisons of plasma homocysteine levels between groups

**FIGURE 1:**
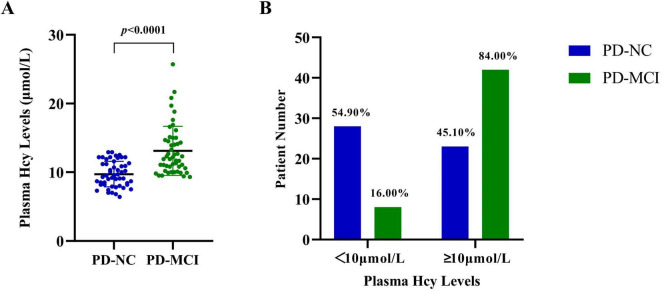
Plasma Hcy levels in PD-NC and PD-MCI patients. **(A)** The plasma Hcy levels of each patient in the PD-NC and PD-MCI groups. **(B)** The proportion of PD-NC and PD-MCI patients having Hcy levels higher or lower than 10 μmol/L. PD-NC, Parkinson’s disease with normal cognition; PD-MCI, Parkinson’s disease with mild cognitive impairment; Hcy, homocysteine.

Although the average Hcy levels of all patients fell within the normal range (11.41 ± 3.30 μmol/L, < 15 μmol/L), the Hcy levels of PD-MCI patients (13.12 ± 3.57 μmol/L) were significantly higher than those of PD-NC patients (9.72 ± 1.86 μmol/L, *p* < 0.0001, Figure 1A). Among the total 65 PD patients with Hcy levels exceeding 10 μmol/L, 42 (64.62%) were in the PD-MCI group, compared to 23 (35.38%) in the PD-NC group. Furthermore, 84.00% of PD-MCI patients had Hcy levels higher than 10 μmol/L, while only 45.10% of PD-NC patients had higher Hcy levels (*p* < 0.0001, Figure 1B). Additional logistic regression analysis was conducted, controlling for confounding factors, to explore the extent of the increased odds associated with elevated Hcy (Table 2). After correcting for age, gender, years of education, and disease duration, each additional one μmol/L of Hcy was associated with a 115% increase in the odds of PD-MCI (OR = 2.15, 95% CI: 1.45∼3.21), and when Hcy levels were ≥ 10 μmol/L, the odds of PD-MCI increased by 471% (OR = 5.71, 95% CI: 1.62∼20.11).

**TABLE 2:** The association of plasma Hcy levels with the odds of PD-MCI.

	Unadjusted		Model 1*		Model 2^#^	
	**OR**	**95% CI**	**OR**	**95% CI**	**OR**	**95% CI**
Hcy	1.85	1.41–2.43	2.15	1.45–3.21	1.85	1.21–2.83
**Hcy level**						
< 10 μmol/L	1.00		1.00		1.00	
≥ 10 μmol/L	6.39	2.51–16.29	5.71	1.62–20.11	2.12	0.50–8.97

*Adjusted with age, sex, education year, and duration of disease.

^#^Adjusted with age, sex, education year, duration of disease, and levodopa-equivalent daily dose. Hcy, homocysteine; OR, odds ratio; CI, confidence interval.

Given the potential homocysteine toxicity resulting from levodopa treatment, correction for LEDD in logistic regression analysis revealed that each additional one μmol/L of Hcy remained associated with an 85% increase in the odds of PD-MCI (OR = 1.85, 95% CI: 1.21∼2.83). When Hcy levels were ≥ 10 μmol/L, the odds of PD-MCI also increased by 112% (OR = 2.12, 95% CI: 0.50∼8.97), though this increase did not reach statistical significance.

### 3.3 Associations between plasma homocysteine levels and cognitive domains or other non-motor symptoms

Further analysis using a generalized linear model identified significant associations between plasma Hcy levels and cognitive domains, as well as other non-motor symptom rating scales (Table 3). Increased Hcy levels were significantly associated with higher scores in BDI (β = 0.806, 95% CI: 0.359∼1.253, *p* = 0.0004), GDS (β = 0.440, 95% CI: 0.053∼0.826, *p* = 0.0257), PDQ-39 (β = 1.826, 95% CI: 0.296∼3.356, *p* = 0.0193), and ESS (β = 0.373, 95% CI: 0.133∼0.613, *p* = 0.0023), as well as longer completion time for the SCWT-C (β = 3.813, 95% CI: 1.662∼5.964, *p* = 0.0005) and TMT-A (β = 2.793, 95% CI: 0.348∼5.238, *p* = 0.0251).

**TABLE 3:** The correlation of plasma Hcy levels with cognitive function or non-motor symptom assessments.

	Unadjusted		Model 1*		Model 2^#^	
	**β (95% CI)**	***p*-value**	**β (95% CI)**	***p*-value**	**β (95% CI)**	***p*-value**
**Non-motor symptom assessment**						
BDI	0.806 (0.359, 1.253)	**0.0004**	0.683 (0.178, 1.188)	**0.0081**	0.735 (0.056, 1.415)	**0.0340**
GDS	0.440 (0.053, 0.826)	**0.0257**	0.384 (−0.049, 0.817)	0.0820	0.189 (−0.380, 0.757)	0.5149
PDQ-39	1.826 (0.296, 3.356)	**0.0193**	1.717 (0.145, 3.289)	**0.0323**	1.295 (−1.027, 3.616)	0.2744
NMSS	0.308 (−0.152, 0.769)	0.1895	0.184 (−0.304, 0.672)	0.4605	−0.096 (−0.842, 0.650)	0.8010
ESS	0.373 (0.133, 0.613)	**0.0023**	0.287 (0.008, 0.567)	**0.0441**	0.605 (0.178, 1.033)	**0.0055**
RBDSQ	0.044 (−0.132, 0.219)	0.6261	−0.058 (−0.236, 0.119)	0.5202	−0.167 (−0.400, 0.066)	0.1593
SSST-12	0.027 (−0.120, 0.174)	0.7181	0.100 (−0.056, 0.256)	0.2069	0.169 (−0.061, 0.398)	0.1491
**Cognitive function assessment**						
MMSE	−0.037 (−0.179, 0.105)	0.6088	0.053 (−0.086, 0.192)	0.4556	0.150 (−0.059, 0.358)	0.1604
**Memory**						
AVLT-Delay	−0.130 (−0.308, 0.048)	0.1534	−0.031 (−0.230, 0.169)	0.7629	0.127 (−0.182, 0.436)	0.4211
AVLT-Total	−0.213 (−0.801, 0.374)	0.4772	0.082 (−0.569, 0.733)	0.8045	0.332 (−0.603, 1.266)	0.4864
CFT-Delay	0.005 (−0.433, 0.442)	0.9837	0.227 (−0.237, 0.690)	0.3378	0.310 (−0.385, 1.005)	0.3821
**Executive function**						
SCWT-C (sec)	3.813 (1.662, 5.964)	**0.0005**	3.227 (0.675, 5.779)	**0.0132**	2.724 (−0.838, 6.285)	0.1339
SCWT-C (score)	−0.472 (−0.971, 0.027)	0.0637	−0.623 (−1.221, −0.025)	**0.0413**	−1.123 (−1.845, −0.401)	**0.0023**
TMT-B (sec)	2.753 (−1.948, 7.454)	0.2511	2.144 (−2.724, 7.012)	0.3880	2.085 (−5.105, 9.274)	0.5698
**Language**						
BNT	−0.067 (−0.372, 0.239)	0.6692	0.068 (−0.231, 0.366)	0.6582	−0.030 (−0.391, 0.330)	0.8694
AFT	−0.189 (−0.437, 0.058)	0.1339	−0.056 (−0.288, 0.176)	0.6338	0.175 (−0.129, 0.479)	0.2580
**Visuospatial function**						
CFT	−0.072 (−0.753, 0.608)	0.8351	0.340 (−0.343, 1.022)	0.3292	0.049 (−0.959, 1.056)	0.9247
CDT	0.128 (−0.335, 0.590)	0.5893	0.201 (−0.327, 0.730)	0.4552	−0.033 (−0.870, 0.804)	0.9386
**Attention/working memory**						
SDMT	−0.345 (−1.237, 0.546)	0.4475	−0.154 (−1.053, 0.745)	0.7373	0.433 (−0.842, 1.707)	0.5057
TMT-A (sec)	2.793 (0.348, 5.238)	**0.0251**	2.924 (0.376, 5.473)	**0.0245**	2.280 (−1.963, 6.522)	0.2923

Plasma Hcy levels were the independent variable, and cognitive function or non-motor symptom assessments were the dependent variables used in the generalized linear models. The bold *p*-value was statistically significant.

*Adjusted with age, sex, education year, and duration of disease.

^#^Adjusted with age, sex, education year, duration of disease, and levodopa-equivalent daily dose. AFT, Animal Fluency Test; AVLT, Auditory Verbal Learning Test; BDI, Beck Depression Inventory; BNT, Boston Naming Test; CDT, Clock Drawing Test; CFT, Rey-Osterrieth Complex Figure Test; ESS, Epworth Sleepiness Scale; GDS, Geriatric Depression Rating Scale; MMSE, Mini-Mental State Examination; NMSS, Non-Motor Symptoms Scale; PDQ-39, 39-item Parkinson’s Disease Questionnaire; RBDSQ, Rapid Eye Movement Sleep Behavior Disorder Screening Questionnaire; SCWT-C, Stroop Color-Word Test C; SDMT, Symbol Digit Modalities Test; TMT-A & B, Trail Making Test A and B; SSST-12, Sniffin’ Sticks Screening 12 Test; Hcy, homocysteine.

After correcting for age, gender, years of education, and disease duration, the associations with BDI, PDQ-39, ESS, SCWT-C completion time, and TMT-A completion time persisted. Hyperhomocysteinemia was negatively associated with the SCWT-C score (β = −0.623, 95% CI: −1.221∼−0.025, *p* = 0.0413). With additional correction for LEDD, only higher scores in BDI (β = 0.735, 95% CI: 0.056∼1.415, *p* = 0.0340) and ESS (β = 0.605, 95% CI: 0.178∼1.033, *p* = 0.0055), along with poorer performance on the SCWT-C (β = −1.123, 95% CI: −1.845∼−0.401, *p* = 0.0023), which reflected executive function, were significantly associated with elevated Hcy levels.

## 4 Discussion

In our study, we noted a substantial increase in plasma homocysteine levels among patients with PD-MCI in comparison to those with PD-NC. Interestingly, even within the normal range (< 15 μmol/L), a comparatively higher Hcy level (≥ 10 μmol/L) was linked to a higher odds of PD-MCI, a relationship that remained significant after adjusting for confounding factors. Our results additionally solidify a connection between plasma Hcy levels and the performance on certain cognitive assessments, particularly those evaluating executive functions in PD.

To date, there have been limited studies exploring the association between Hcy and PD-MCI, yielding disparate findings. Rodriguez-Oroz et al. (2009) investigated the role of Hcy among PD-NC, PD-MCI and PDD patients, reporting no significant differences in Hcy levels among these cognitive statuses and no correlation between plasma Hcy levels and performance on any neuropsychological tests. Similarly, Licking et al. (2017) also found no differences in plasma Hcy levels between PD-NC, PD-MCI and PDD patients, however, their results revealed a notable association between plasma Hcy and specific cognitive domains, including language, memory and executive function, as assessed by semantic verbal fluency task, Hopkins verbal learning-delayed test, and digital symbol test. It is worth noting that the participants in these studies were older (mean age of ∼70 years old) and had longer disease duration (mean duration of 10–14 years), which may differ from our cohort. In contrast, a study by Kobak Tur and Ari (2023) enrolled younger PD patients with shorter disease duration. They found higher Hcy levels in PD-MCI patients and revealed a significant association between Hcy levels and visuospatial/executive function using the Montreal Cognitive Assessment Scale (MoCA). These results align with our findings and suggest a specific association between executive function impairment in PD-MCI patients and elevated Hcy levels, as indicated by different neuropsychological tests. Furthermore, a literature review was conducted on the association between Hcy and impaired cognitive domains in PD, even if some studies did not specifically focus on PD-MCI (Table 4). In all studies with positive results, executive function has been found to be associated with plasma Hcy levels, indicating that executive function may be a characteristically affected cognitive area in PD patients with elevated Hcy levels.

**TABLE 4:** Summary of the literature about the association between Hcy and impaired cognitive domains in PD.

References	Patient number	Age (year)	Disease duration (year)	H & Y stage	Plasma Hcy level (μ mol/L)	Results	Cognition assessment
Ozer et al., 2006	PD-hiHcy* = 17, PD-loHcy = 22	PD-hiHcy = 68.5 ± 8.8, PD-loHcy = 64.4 ± 9.5	PD-hiHcy = 6.4 ± 3.3, PD-loHcy = 6.4 ± 4.3	N.A.	PD-hiHcy = 21.9 ± 11.2, PD-loHcy = 10 ± 2.6	Memory, visuospatial, and executive function are correlated with Hcy, but no general cognitive difference between the two groups	Multiple neuropsychological tests
Hassin-Baer et al., 2006	PD-hiHcy = 25, PD-mHcy = 24, PD-loHcy = 23	PD-hiHcy = 69.3 ± 11.4, PD-mHcy = 67.0 ± 11.4, PD-loHcy = 69.9 ± 12.3	PD-hiHcy^#^ = 8 (6–11), PD-mHcy = 6 (4–10.8), PD-loHcy = 5 (3–7)	N.A.	PD-hiHcy > 16.7, PD-mHcy = 12.5–16.7, PD-loHcy < 12.5	No any cognitive differences among the three groups	Multiple neuropsychological tests
Rodriguez-Oroz et al., 2009	PD-NC = 37, PD-MCI = 22, PDD = 30	PD-NC = 69.97 ± 6.5, PD-MCI = 70.23 ± 5.2, PDD = 74.87 ± 6.15	PD-NC = 14.68 ± 4.62, PD-MCI = 13.05 ± 3.69, PDD = 14.73 ± 4.45	PD-NC = 3.35 ± 0.91, PD-MCI = 3.43 ± 0.62, PDD = 3.94 ± 0.52	PD-NC = 14.9 ± 4.7, PD-MCI = 15.1 ± 4.3, PDD = 15.4 ± 5.4	No correlation was found between Hcy levels and cognitive performance	Multiple neuropsychological tests
Licking et al., 2017	PD-NC = 56, PD-MCI = 175, PDD = 63	All = 68.0 ± 9.1	All = 9.95 ± 6.8	All^#^ = 2.5 (IQR = 2–3)	No significant differences among the three groups	Language and memory function are correlated with Hcy, and less clear of executive function	Multiple neuropsychological tests
Kobak Tur and Ari, 2023	PD-NC = 25, PD-MCI = 36	PD-NC = 57.8 ± 12.09, PD-MCI = 66.42 ± 10.75	PD-NC = 4.6 ± 2.38, PD-MCI = 6.67 ± 3.82	PD-NC = 1.86 ± 0.6, PD-MCI = 2.4 ± 0.66	PD-NC = 13.34 ± 5.31, PD-MCI = 15.4 ± 4.32	Visuospatial/executive function is correlated with Hcy	MoCA
Our study	PD-NC = 51, PD-MCI = 50	PD-NC = 62.37 ± 10.14, PD-MCI = 62.82 ± 10.27	PD-NC = 3.58 ± 3.29, PD-MCI = 4.71 ± 3.99	PD-NC = 2.30 ± 0.71, PD-MCI = 2.49 ± 0.71	PD-NC = 9.72 ± 1.86, PD-MCI = 13.12 ± 3.57	Executive function is correlated with Hcy	Multiple neuropsychological tests

*PD-hiHcy represented patients with plasma Hcy levels higher than 14 μmol/L in this study, and PD-loHcy represented patients with Hcy levels lower than 14 μmol/L.

^#^Disease duration and H & Y stage in corresponding studies were represented as median (interquartile range). Other data in the table were expressed as the means ± standard deviation. PD-NC, Parkinson’s disease (PD) with normal cognition; PD-MCI, PD with mild cognitive impairment; PDD, PD with dementia; PD-hiHcy, PD patients with high Hcy levels; PD-mHcy, PD patients with median Hcy levels; PD-loHcy, PD patients with low Hcy levels; H & Y stage, Hoehn & Yahr-staging-scale; Hcy, homocysteine; MoCA, Montreal Cognitive Assessment; N.A., no answer.

Some imaging and metabolomics studies have also explored the role of Hcy in the brains of PD patients. Sampedro et al. (2022) identified a correlation between elevated Hcy levels and cognitive impairment in PD, along with thinning of the frontal cortex and microstructural damage. Additionally, some metabolomics analyses revealed accumulation of Hcy in the frontal cortex of PD subjects, emerging as a prominent characteristic associated with dementia (Kalecký et al., 2022; Kalecký and Bottiglieri, 2023). Given the critical role of the fontal cortex in executive function, the specific targeting of Hcy to the frontal cortex might support the hypothesis that executive function impairment is associated with elevated Hcy levels in PD patients.

As previously mentioned, the variability in age and disease duration among enrolled PD patients in different studies may partially explain the discrepancies in results regarding the association between Hcy and cognitive impairment. Cognitive decline in PD patients with advanced age and longer disease duration could be influenced by numerous factors beyond Hcy, such as aging, the progressive accumulation of Aβ and α-synuclein pathology in the cortex, and the development of motor symptoms (Aarsland et al., 2021). The effect of these confounding factors could gradually intensify and ultimately become the primary contributors to cognitive damage, potentially overshadowing the direct association between Hcy and cognitive impairment. Clinical trials of B-vitamin treatment for hyperhomocysteinemia and cognitive decline have also emphasized that the cognitive benefit of B-vitamin therapy might be expected primarily among patients who are not yet in an advanced disease stage (Ahlskog, 2023). Furthermore, it’s noteworthy that the threshold for defining hyperhomocysteinemia in other studies (> 14 or 15 μmol/L) was considerably higher than that used in our studies (> 10 μmol/L). Although some clinical trials of B-vitamin therapy found cognitive benefits only in subjects with high baseline Hcy levels (de Jager et al., 2012), the results of our study indicate that relatively higher levels of Hcy, even within the normal range, are strongly associated with cognitive impairment in PD patients.

Our study has revealed a significant correlation between elevated levels of Hcy and depression, as well as excessive daytime sleepiness. Depression stands out as the most prevalent emotional disorder among PD patients, with an approximate prevalence rate of 40% (Cummings, 1992). Some studies have found significantly higher incidences of mood symptoms, such as apathy, anxiety, and depression, in older adults with MCI compared to those with normal cognition (Geda et al., 2008). Conversely, mood symptoms have been linked to a heightened risk of subsequent MCI development (Geda et al., 2014). Additionally, previous research has suggested that depression might serve as an early indicator of cognitive impairment, sharing certain neuropathological features with MCI and dementia (Panza et al., 2010). This notion is substantiated by the common impairment of frontal-related cognitive function in PD-MCI and depressive patients (Lockwood et al., 2002; Kalbe et al., 2016). Studies have suggested that elevated Hcy level is an independent risk factor for depression in the general population (Moradi et al., 2021), as well as in PD patients (O’Suilleabhain et al., 2004). Our study found that elevated Hcy levels were associated not only with cognitive dysfunction in PD-MCI but also with increased score on the BDI, suggesting shared neuropathogenesis that may be partially explained by frontal-targeting impairment associated with elevated Hcy levels. Excessive daytime sleepiness has been reported to be related to cognitive decline in PD (Xiang et al., 2019). Kobak Tur and Ari (2023) also described an inverse correlation of ESS scores with MoCA in PD-MCI patients. Furthermore, a cross-sectional study of the general population in China has reported an association of excessive daytime sleepiness with hyperhomocysteinemia (Zhang et al., 2016). Although we described the significant paralleled relationship between excessive daytime sleepiness and elevated Hcy levels in PD patients, further studies are required to validate whether sleepiness is caused by the direct effect of Hcy or an incidental alteration associated with worsened cognition status.

To date, the causal relationship between elevated Hcy levels and cognitive decline in PD remains undetermined. One limitation of our study is that while we have adjusted for many potential confounders presented before, we cannot exclude the possible influence of unmeasured confounders, such as various comorbidities, including chronic inflammation and metabolic diseases (e.g., diabetes, hypertension, coronary artery disease, dyslipidemia), which could significantly be associated with Hcy levels. Another limitation of our study is its cross-sectional nature, which makes it difficult to establish evidence for a causal directionality between PD-MCI and hyperhomocysteinemia. Prospective follow-up of cognitive impairment progression in these patients would aid in clarifying the pathogenic effect of baseline Hcy levels on cognitive deterioration, particularly in early PD cases. A prospective cohort study by Sleeman et al. (2019) revealed that higher baseline Hcy concentrations could predict a decline in MoCA scores over a 54-month follow-up period in newly diagnosed PD patients. Additionally, in certain clinical trials investigating B-vitamin treatment for hyperhomocysteinemia, MRI documentation demonstrated a significantly reduced brain atrophy rate over 24 months, particularly in the top quartile of baseline Hcy levels (Smith et al., 2010). Furthermore, cognitive benefit from B-vitamin therapy were observed solely in participants with elevated baseline Hcy levels (de Jager et al., 2012). All these interconnecting results seemingly indicate a critical role of Hcy in the pathogenesis of cognitive impairment in the general population and PD patients. Although the specific molecular mechanism is still debated and confusing, some well-established mechanisms about the neurotoxicity of Hcy, such as inhibiting neurotransmitters, increasing oxidative stress and mitochondrial dysfunction, promoting inflammation, and inducing neuronal apoptosis, were revealed by dozens of basic research studies (Bhatia and Singh, 2015; Zhou, 2023). A recently published animal study reported that aging and L-methionine administration may increase brain Hcy levels in mice, leading to elevated homocysteinylation of α-synuclein, which facilitated its fibrillization, seeding capacity, and neurotoxicity, exacerbating α-synuclein pathology in the cortex of a mouse model of PD. In contrast, blocking α-synuclein homocysteinylation could ameliorate the toxicity of Hcy *in vivo* (Zhou et al., 2023). This result may provide an additional evidence of a causal relationship between Hcy and cognitive impairment from the perspective of α-synuclein pathology in the cortex.

## 5 Conclusion

Our research findings reveal a significant association between plasma Hcy levels and PD-MCI, particularly concerning executive dysfunction, even within a normal range of Hcy levels (< 15 μmol/L). These results suggest that Hcy might represent a potentially treatable risk factor and an interesting target for future intervention studies aimed at preventing cognitive impairment in PD. Routine screening of plasma Hcy levels and cognitive function in early PD patients might be beneficial in this regard.

## Data Availability

The original contributions presented in this study are included in this article/supplementary material, further inquiries can be directed to the corresponding authors.
